# ZHX2 in health and disease

**DOI:** 10.3389/fonc.2022.1038890

**Published:** 2022-11-18

**Authors:** Na Li, Zhuanchang Wu, Chunhong Ma

**Affiliations:** ^1^ Key Laboratory for Experimental Teratology of Ministry of Education and Dept. Immunology, School of Basic Medical Sciences, Cheeloo Medical College, Shandong University, Jinan, Shandong, China; ^2^ Key Laboratory of Infection and Immunity of Shandong Province, Shandong University, Jinan, Shandong, China

**Keywords:** ZHX2, tumor repressor, oncogene, cell differentiation, lipid metabolism, immunoregulation

## Abstract

As a transcriptional factor and the negative regulator of alpha fetal protein (AFP), Zinc fingers and homeoboxes 2 (ZHX2) has a well-established role in protection against hepatocellular carcinoma (HCC). However, recent studies have suggested ZHX2 as an oncogene in clear cell renal cell carcinoma (ccRCC) and triple-negative breast cancer (TNBC). Moreover, mounting evidence has illustrated a much broader role of ZHX2 in multiple cellular processes, including cell proliferation, cell differentiation, lipid metabolism, and immunoregulation. This comprehensive review emphasizes the role of ZHX2 in health and diseases which have been more recently uncovered.

## Introduction

ZHX2, a member of the ZHX (Zinc fingers and homeoboxes) family, is a ubiquitous transcriptional factor that was first identified as a negative regulator of murine postnatal alpha fetal protein (AFP) (1). In 1977, Roushlatti and colleagues compared serum AFP in different mouse strains and found a gene which they called *Regulator of Alpha-fetoprotein* (*Raf*), subsequently renamed *Alpha-fetoprotein regulator 1* (*Afr1*), negatively regulated the AFP expression in adult mice (1, 2). In 2005, Perincheri et al. further refined and identified *Zhx2* as the homologous gene of *Afr1* by positional cloning (3). Human *ZHX2* was first cloned by Nagase et al. from a size-fractionated brain cDNA library in 1998 (2). In 2003, human ZHX2 was then identified as a ZHX1-interacting protein by Kawata et al. (4).

ZHX2 has been extensively studied in cancer development. ZHX2 suppresses the transcription of oncofetal genes *AFP (* 1, 3, 5) and *glypican 3* (*GPC3*), and works as a tumor suppressor gene in HCC (5, 6). Subsequent studies have found that ZHX2 is widely expressed and participates in many types of cancer. Consistent with findings in HCC, low ZHX2 expression correlates with poor prognosis of thyroid cancer (7), multiple myeloma (8–10), and chronic lymphocytic leukemia (11, 12). On the contrary, ZHX2 promotes the development of ccRCC (13–15), TNBC (16), and gastric cancer (17, 18). Beyond regulating cancer development, the latest reports have shown that ZHX2 involves in several other physiological or pathological processes, including cell differentiation and development (19–21), lipid metabolism (22–24), and viral replication (25, 26). Especially, ZHX2 is abundantly expressed in the thymus and spleen (2) and there is clear evidence supporting the involvement of ZHX2 in regulating B cell development (27), NK cell maturation (28), and macrophage polarization (29–31).

In this review, we outline these new advances in ZHX2 mediated regulation in health and diseases. We also discuss the multiple mechanisms involved in controlling ZHX2 expression and transcription.

## ZHX2 protein structure and its role as a transcription factor

The human *ZHX2* gene is localized on chromosome 8q24.13 and consists of 4 exons (4). The third exon is the sole coding exon of *ZHX2* which encodes a protein of 837 amino acid residues (4). Human ZHX2 protein, like the other two family members ZHX1 and ZHX3, contains two Cys-Xaa_2_-Cys-Xaa_12_-His-Xaa_4_-His-type zinc finger domains (Znf) and four homeodomains (HD) (originally thought as five HDs) (4). Besides, ZHX2 contains a proline-rich region (PRR) at position 408 to 440 between HD1 and HD2 (4). The homology of ZHX2 protein in humans and mice is as high as 87%. Kawata et al., in 2003, identified ZHX2 as a ubiquitous transcription factor. ZHX2 interacts with nuclear transcription factor Y subunit alpha (NF-YA) and forms homodimers or heterodimers with ZHX1 or ZHX3 to exert transcriptional inhibitory function (5). The amino acid sequence between residues 195 and 358 containing HD1 is required for homodimerization of ZHX2, and ZHX2 interacts with NF-YA *via* the region between 263 and 497 residues (4). Similar to full-length ZHX2, truncated ZHX2 containing residues 242-446 (ZHX2(242-446)) but not ZHX2(242-439) maintain the capability to localize in the nuclei and suppress the expression of Cyclin A/E in HCC (6). The decreased nucleic ZHX2 expression significantly correlates with poor survival of HCC patients (6). However, how ZHX2 loses its nuclear localization is completely unknown. More studies are required to define the exact nuclear localization signal (NLS) and the molecules or mechanisms regulating the nuclei translocation of ZHX2.

A growing number of genes have been identified as the ZHX2 targets, most of which are cancer-related. ZHX2 not only negatively controls the transcription of liver cancer marker genes *AFP* and *GPC3*, but also inhibits cell proliferation-related genes such as *Cdc25 (* 4)*, Cyclin A/E (* 6), and *Notch1 (* 32). In addition, ZHX2 represses transcription of *multidrug resistance mutation 1* (*MDR1*) (33), *lipoprotein lipase* (*LPL*) (34), *lysine demethylase 2A* (*KMD2A*) (35), and *S100 calcium binding protein A14* (*S100A14*) (7) in HCC and thyroid cancer cells. Although ZHX2 was originally reported to be a ubiquitous transcriptional repressor, recent reports uncover another face of ZHX2 as a transcriptional activator (36, 37). Jiang et al. found that Zhx2 binds *Mup* promoters and is required for high levels of Mup expression in adult mouse liver (36). ZHX2 also binds to the promoter of *phosphatase and tensin homolog (PTEN)* and subsequently promotes the transcription of *PTEN (* 37). Strikingly, several non-coding RNAs have been elucidated as the ZHX2 targets, either enhanced or inhibited. ZHX2 represses transcription of *H19 (* 3, 38, 39), the first imprinted non-coding transcript to be identified. In glioma cells, ZHX2 binds to the promoter region of *linc00707* and negatively regulates its expression, leading to glioma cells proliferation, migration and invasion, and vasculogenic mimicry (VM) formation (40). On the contrary, ZHX2 increases transcription of *miR-24-3p* and *linc01431*, which targets *SREBP1c (* 24) and PRMT1 (26) in hepatocytes respectively.

The mechanism by which ZHX2 controls target gene transcription is not fully understood. ZHX2 was originally known as an NF-YA interacting protein (4) and therefore represses transcription of *MDR1*, *Cdc25*, and *Notch1* by interacting with NF-YA (4, 6, 32, 33). However, there is no evidence for the presence of NF-YA binding sites in promoter of some other ZHX2-targeted genes, such as *Cyclin E*, or *AFP (* 5, 6). A global analysis of Zhx2 targets using ChIP-seq in a murine macrophage cell line shows a significant overlap with two known apoptosis regulators Jun (41) and Bcl6 (42), which suggest a strong involvement of Zhx2 in cell apoptosis (30). In ccRCC, ChIP-seq data indicate that the genome-wide chromatin occupancy of ZHX2 overlaps with 75% of p65-binding motifs (13). ZHX2 and RelA/p65 overlapping sites also display a strong enrichment for H3K4me3 and H3K27ac, indicating that ZHX2 colocalizes with NF-κB to active gene promoters (13). In TNBC, the integrated ChIP-seq and gene expression profiling show that ZHX2 and HIF1α co-occupy transcriptional active promoters to promote gene expression (16). These studies suggest that ZHX2 may mainly serve as a transcriptional cofactor, interacting with different coactivators/repressors in different physiological circumstance to control its localization in the genome and downstream transcriptional activity. In addition, the Znf domains of ZHX2 process potential DNA-binding activity, however, whether ZHX2 can bind DNA directly and its consensus binding motif still need to be investigated.

## Control of ZHX2 expression

**Figure 1:**
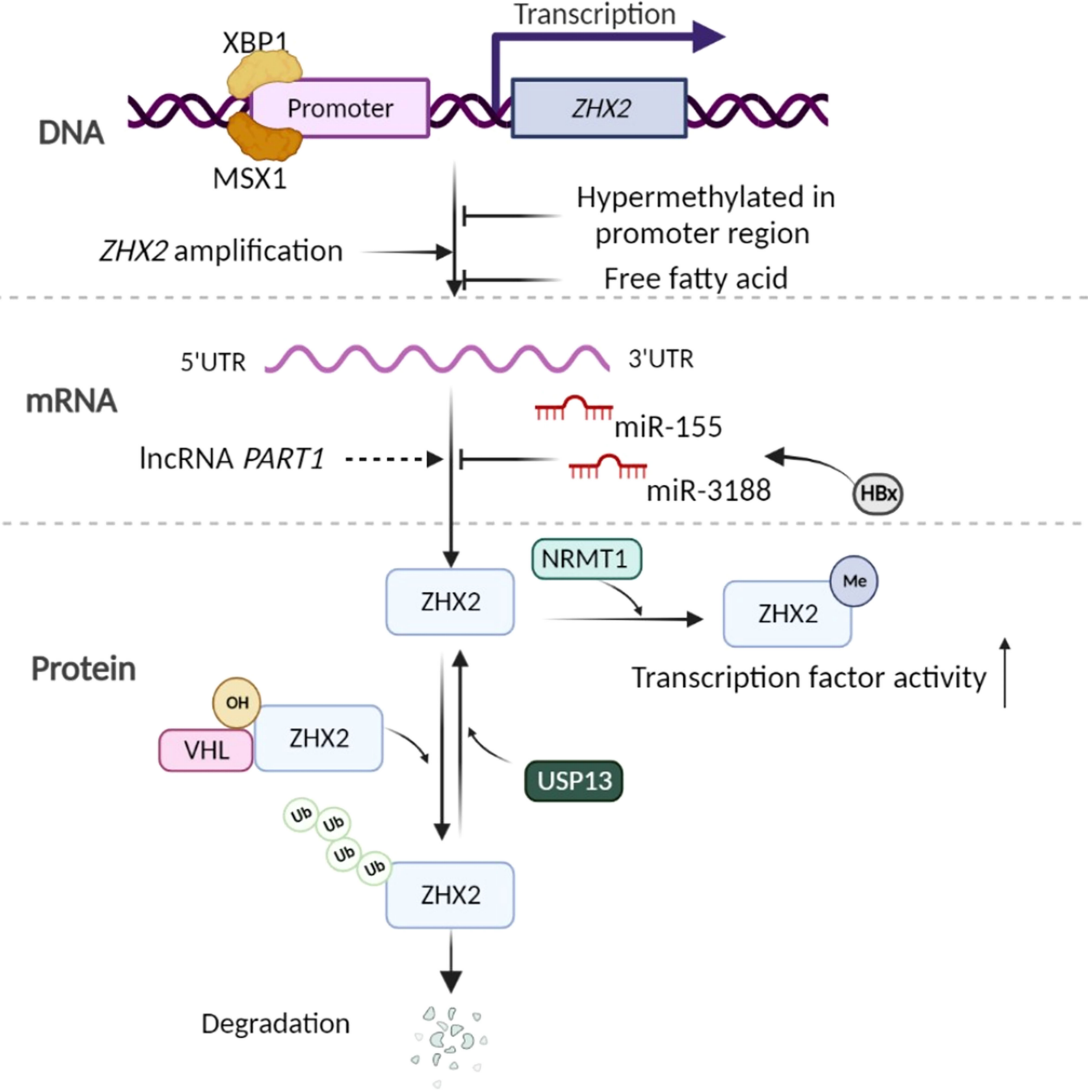
Control of ZHX2 expression. At the gene and transcription level, some transcription factors, hypermethylation of *ZHX2* promotor, and cellular stimuli such as free fatty acid are known to regulate *ZHX2* transcription. Concurrently, *ZHX2* gene amplification contributes to its enhanced expression in cancer. At the post-transcription level, miR-155 and miR-3188 upregulated by HBx inhibit *ZHX2* mRNA translation, but lncRNA PART1 promotes *ZHX2* mRNA level by altering the miRNA landscape. At the PTMs level, hydroxylated ZHX2 protein is recognized and degraded by E3 ubiquitin ligase VHL, which is inhibited by USP13-induced deubiquitination, while NRMT1-mediated Nα-methylation of ZHX2 promotes its transcription factor activity. Created using Biorender.com.

ZHX2 expression is tightly regulated under different circumstances. A computational network study indicates ZHX2 as one of the most regulated transcription factors in myeloid cells to avoid an avalanche of transcriptional events (31). In Hodgkin lymphoma (HL), a chromosomal rearrangement far upstream region of *ZHX2* gene results in the transcriptional silence of *ZHX2*, and two transcription factors, homeodomain protein MSX1 and bZIP protein XBP1, are identified to directly regulate ZHX2 expression (11). Furthermore, human ZHX2 is lower expressed in fetal liver, increased after birth, and silenced in HCC (43–45). Consequently, multiple mechanisms are revealed to control ZHX2 expression at different levels (**Figure 1**):

*At the ZHX2 gene transcription level*- Lv et al. found that *ZHX2* promoter region is hypermethylated in HCC, suggesting that the hypermethylation-mediated silencing of *ZHX2* is an epigenetic event involved in HCC (45). In addition, copy number analysis showed that *ZHX2* gene is amplified in various cancers, including ovarian cancer (~40%) and breast cancer (~15%). The *ZHX2* copy number significantly correlates with enhanced ZHX2 expression (16). Wu et al. (34) and Zhao et al. (37)found that Zhx2 expression can be repressed by free fatty acid in hepatocytes. Constantly, hepatic Zhx2 is reduced in mice with fatty liver, indicating that ZHX2 could be regulated by the metabolic microenvironment. This is consistent with a previous computational network study indicating ZHX2 as one of the most regulated transcription factors in myeloid cells (31). The detailed mechanisms regulating ZHX2 expression in different circumstances need to be further studied.

*At the post-transcription level*- microRNAs (miRNAs) are short non-coding RNAs that regulate gene expression post-transcriptionally. They generally bind to the 3’-UTR (untranslated region) of their target mRNAs and reduce protein production by destabilizing mRNA or translational silencing (46, 47). HBV-encoded proteins, particularly a well-known oncogenic protein HBx, drive the high expression of miR-155, which binds to seed sites in the 3’-UTR of the *ZHX2* mRNA and inhibit its translation (48). Similarly, HBx promotes CREB-mediated activation of miR-3188 to repress ZHX2 expression, leading to activated Notch signaling in HCC (32). While in TNBC, lncRNA *PART1* promotes *ZHX2* transcription (49).

*At the posttranslational modifications (PTMs) level*- Zhang et al. report that inactivation of the von Hippel-Lindau (VHL) E3 ubiquitin ligase in ccRCC leads to the accumulation of ZHX2 protein and its nuclear localization. ZHX2 protein hydroxylation at proline 427, 440, and 464 allows VHL to bind and promote its protein degradation (13). However, a deubiquitinase USP13 inhibits the ubiquitination of ZHX2 and enhances its stability (15). A recent study found that the N-terminal methylation (Nα-methylation) of ZHX2 by the methyltransferase NRMT1 regulates its transcription factor activity and its occupancy on targeted promoters (50). Up to now, whether there are other PTMs and their roles in ZHX2 trafficking, stability, and transcriptional activity are less clear.

## ZHX2 in cancer-a context-dependent tumor repressor or driver?

**Figure 2:**
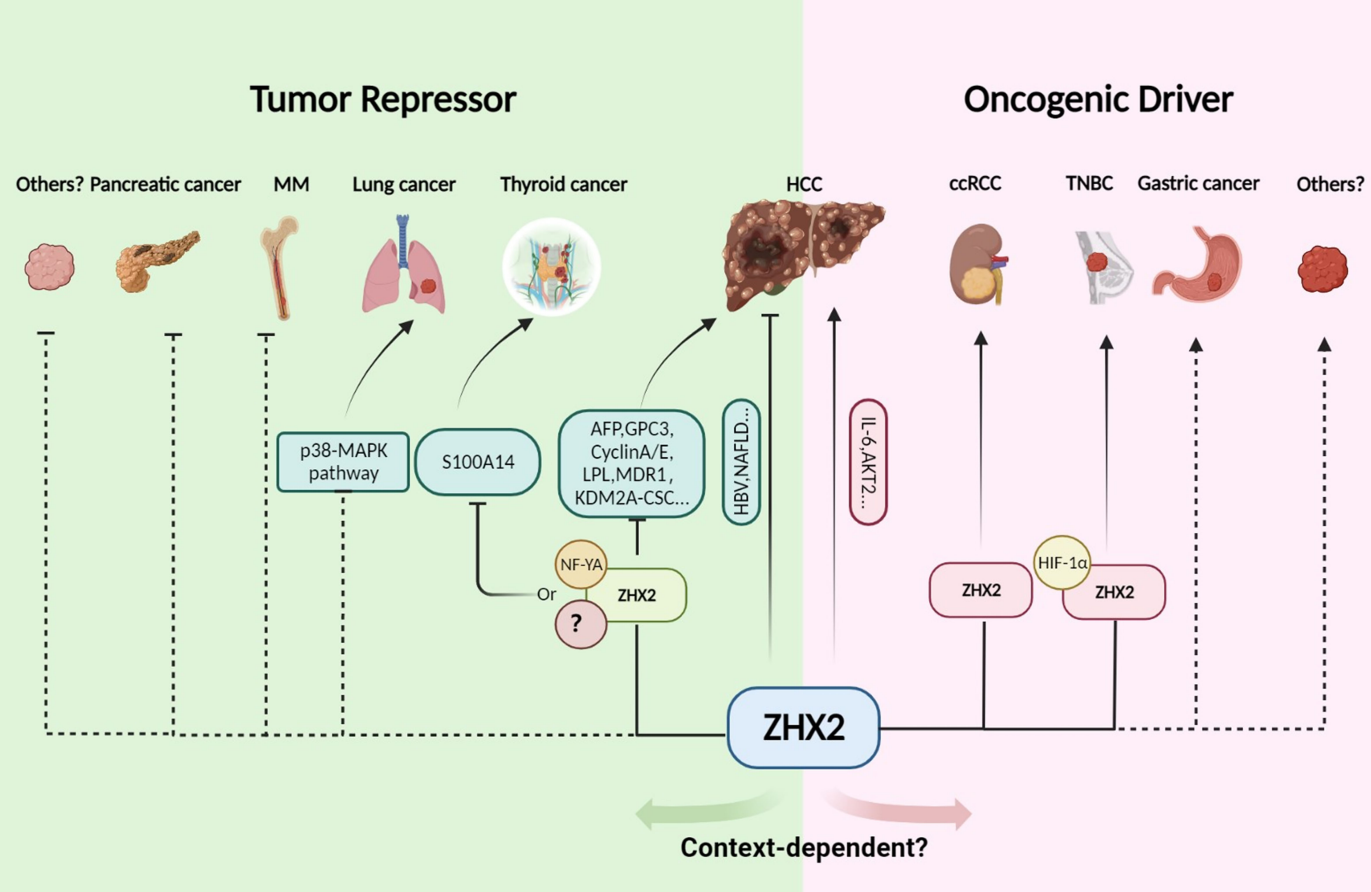
The tumor repressor or driver role of ZHX2 in cancer. In HCC, ZHX2 has a context-dependent role. ZHX2 inhibits HCC *via* multiple mechanisms, but whole body knockout of *Zhx2* reduces DEN-induced liver tumors indicating its complex roles. In HCC, lung cancer, multiple myeloma, HL, and thyroid cancer, ZHX2 acts as a tumor suppressor and transcriptionally represses AFP, GPC3, Cyclin A/E, LPL, KDM2A, and S100A14 expression *via* interacting with NF-YA or other unknown partners to restrict cancer progress. However, in ccRCC and TNBC, ZHX2 plays an oncogenic driver role by interacting with p65 and HIF1α to activate oncogenic signaling. Created using Biorender.com.

ZHX2 is initially identified as an AFP repressor and a tumor repressor in HCC (3, 5). Whereafter, abnormal expression of ZHX2 is reported in multiple types of tumor (6, 8, 11). Furthermore, ZHX2 expression is closely related to the malignancy and poor prognosis of B-cell lymphoma (11, 12), myeloma (8–10), lung cancer (51), and thyroid cancer (7), suggesting that ZHX2 plays an important role in tumorigenesis and cancer development. Interestingly, latest studies reported that ZHX2 functions as an oncogene in ccRCC (13, 14) and TNBC (16). Likewise, Jiang et al. reported that the whole-body knockout of *Zhx2* results in reduced liver tumors in diethylnitrosamine (DEN)-induced HCC mice (52). Therefore, ZHX2 is abnormally expressed in multiple tumors and plays different roles, either acting as a tumor suppressor or oncogene in a context-dependent manner (**Figure 2**). Here, we outline the role of ZHX2 in multiple tumors.

### ZHX2 as a tumor suppressor in HCC and other cancers

ZHX2 regulates the posttranscriptional silencing of oncofetal genes *AFP*, and *GPC3*, both of which are expressed in fetal liver, silenced after birth, and reactivated in HCC (43–45). These suggest that ZHX2 contributes to hepatocarcinogenesis as a tumor suppressor. Consistently, our previous study showed that the nuclear ZHX2 is reduced in human HCC tissues compared with adjacent nontumor tissues and nuclear ZHX2 represses HCC cell growth by inhibition of cell cycle genes (*Cyclin A* and *Cyclin E*), demonstrating for the first time the tumor suppressor activity of ZHX2 in HCC (6). In accordance, another study detected the hypermethylation of *ZHX2* promoter and the silencing of ZHX2 expression in HCC tissues (45). Subsequent studies further illustrated the critical role of ZHX2 as a tumor suppressor in HCC with a variety of etiologies, including NASH-related HCC (34, 37) and HBV-related HCC (25, 32). However, there is conflicting data on the role of ZHX2 in HCC. Hu et al. reported increased ZHX2 staining in HCC tissues and higher ZHX2 expression in poorly differentiated and metastasis samples, indicating that ZHX2 might promote HCC progression (53). Jiang et al. recently showed that whole body *Zhx2* knockout (*Zhx2^KO^*) leads to dramatically reduced liver cancer in DEN-induced HCC mouse model, indicating the oncogenic role of ZHX2 in DEN-induced liver tumor model (52). Interestingly, compared with *Zhx2^KO^* mice, DEN induces more tumors in liver-specific *Zhx2* knock-out mice (*Zhx2^Δliv^*) (52). These data suggest that ZHX2 expression in non-parenchymal cells plays a critical role in liver carcinogenesis. Therefore, although most studies support the conclusion that ZHX2 works as a tumor suppressor in HCC, the exact role of ZHX2 in HCC needs to be further defined and ZHX2 expression in non-parenchymal cells should be deeply investigated.

The tumor suppressor role of ZHX2 has also been demonstrated in many other types of tumors including hematological tumors and solid tumors. Spectral karyotyping identified chromosomal rearrangement far upstream region of *ZHX2* gene in Hodgkin lymphoma and this aberration results in ZHX2 silencing (11, 12). Low ZHX2 is associated with poor prognosis in chronic lymphocytic leukemia and multiple myeloma (MM) (8, 54), while higher *ZHX2* mRNA correlates with better overall survival in patients with breast cancer (55) and thyroid cancer (7). ZHX2 inhibits proliferation and promotes apoptosis of lung cancer cells by inhibiting the p38-MAPK signaling pathway (51). Integrative bioinformatics analyses reveal that a miRNA-related SNP (rs3802266-G), which creates a stronger binding site for miR-181-a-2-3p in 3’UTR of *ZHX2* mRNA and consequently reduces ZHX2 expression, was significantly associated with increased risk of pancreatic cancer (56).

ZHX2 not only inhibits tumor growth but also suppresses tumorigenesis and tumor development through multiple mechanisms. Cancer stem cells (CSCs) are critical determinants of tumor relapse and therapeutic resistance (57). ZHX2 counteracts liver cancer stem cell traits by inhibiting KDM2A-mediated demethylation of H3K36 at the promoter region of stemness-associated transcription factors, such as NANOG, SOX2, and OCT4 (35). Furthermore, ZHX2 inhibits thyroid cancer metastasis (7) and is responsible for reduced chemotherapy resistance in HCC (33). ZHX2 enhances the cytotoxicity of anti-cancer drugs in HCC *via* transcriptional repression of MDR1 leading to decreased drug efflux (33). Consistently, a clinical study shows a positive correlation between high ZHX2 expression and longer survival in MM patients (8). However, a recent *in vitro* study shows that treatment of proteasome inhibitor bortezomib (BTZ) leads to enhanced ZHX2 expression which in turn promotes BTZ resistance in cultured MM cells (58). All these data reveal a widespread restriction role of ZHX2 in tumor development at multiple dimensions, including tumor cell proliferation, metastasis, stemness, and chemotherapeutic resistance.

### Oncogenic role of ZHX2 in ccRCC, TNBC, and other tumors

Despite the apparent tumor repression role of ZHX2 in HCC and other cancer types, a number of studies have illustrated that ZHX2 can function as an oncogene. Recently, Zhang et al. reported in Science that the loss of tumor suppressor gene VHL in ccRCC leads to the accumulation of ZHX2 protein in the nuclear, which is correlated with poor survival in patients (13, 59). Mechanistically, ZHX2 interacts with RelA/p65 and promotes oncogenic signaling at least partially *via* activating NF-κB signaling (13). ChIP-seq and gene expression profiling show that 75% of p65 binding sites overlap with those of ZHX2 and their overlapping sites display a strong enrichment of H3K4me3 and H3K27ac (13). In addition, Zhu et al. reported that ZHX2 promotes cell growth and migration through activating MEK/ERK pathway and mediates Sunitinib resistance by regulating the autophagy in ccRCC (14). A similar phenomenon is found in studies of multiple osteosarcoma and gastric cancer (17, 18), where high expression of ZHX2 shows a significant correlation with poor survival. Further, a recent study clarified that ZHX2 functions as a cofactor of the HIF1α to promote HIF1α oncogenic signaling in TNBC (16).

Together, accumulated data demonstrate the critical role of ZHX2 in cancer, either as a tumor suppressor or as an oncogene. However, the detailed mechanism underlying the context-dependent role of ZHX2 in tumors remains largely unknown. Further investigation is required to define the genetic and environmental contexts that influence ZHX2 interaction networks and put genetic interaction networks into different tumors context.

## Beyond cancer — other biological roles for ZHX2

**Figure 3:**
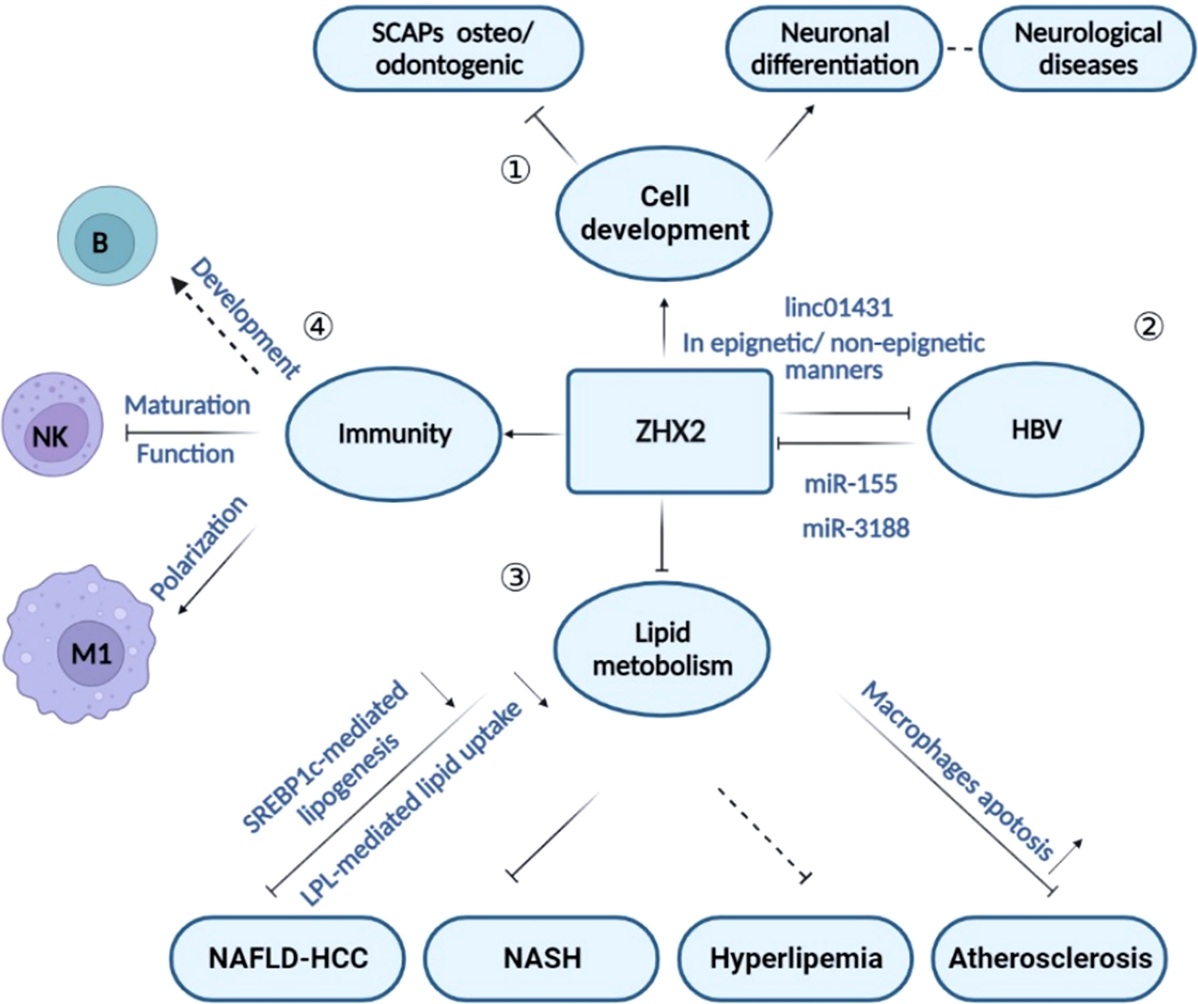
The role of ZHX2 in different physiological and pathological processes. ① Through regulating cell development, ZHX2 is implicated in inhibiting neuronal differentiation and promoting osteo/odontogenic differentiation of stem cells from SCAPs. ② ZHX2 restricts HBV replication *via* CBP/p300 and linc01431-mediated epigenetic repression or *via* inhibiting viral promoter activity in non-epigenetic manners. However, HBx protein reduces ZHX2 expression by upregulating miR-155 and miR-3188 expression. ③ ZHX2 is a critical regulator in lipid hemostasis and plays roles in atherosclerosis, NASH, and NAFLD-HCC progress. ④ ZHX2 is involved in immune regulation by influencing the development of multiple immune cell subsets.

Besides the complicated roles in tumors, recent studies suggest the involvement of ZHX2 in the regulation of cell differentiation, HBV replication, lipid homeostasis, and immune responses (**Figure 3**). In agreement, ZHX2 has been reported in the occurrence of chronic hepatitis B (CHB) (32, 48), metabolism-related diseases (30, 37), nerve-related diseases (19, 60, 61), and immune-related diseases (29) (**Figure 3**). We will discuss the role of ZHX2 in different physiological and pathological processes here.

### ZHX2 in development

The first evidence indicating the involvement of ZHX2 in development comes from the critical role of Zhx2 in the postnatal repression of *Afp* and *Gpc3* in mice (3). In agreement, the dynamic expression of hepatic Zhx2 has been found during liver development and after hepatectomy (21). Zhx2 is low in fetal liver and increases after birth, while Zhx2 expression is significantly declined 24 hours after hepatectomy and then reverses to normal level (21). Therefore, ZHX2 might be a potential therapeutic target in different liver diseases which cause liver injury.

Several studies have illustrated the participation of ZHX2 in regulation of cell development of different origins, such as neurons, blood cells, and bipolar cells. Altered ZHX2 expression has been detected during erythroid differentiation (62) and B cell development (27). Concurrently, ZHX2 is responsible for macrophage polarization (29) and NK cell’s terminal maturation (28). In the nervous system, ZHX2 interacts with Ephrin-B and regulates neural progenitor maintenance (19). Genome-wide analyses identified inherited CNVs (copy number variations) that affect non-genic intervals upstream *ZHX2* in autism spectrum disorder (ASD) patients (61). Exome sequencing in subjects with familial corticobasal degeneration (CBD) shows that mutations in *ZHX2* gene may cause its structural changes, indicating the possible involvement of ZHX2 in corticobasal degeneration (63). In the process of tooth root development, ZHX2 knockdown reduces the mineralization of stem cells from the apical papilla (SCAPs) and promotes SCAPs proliferation (20). Also, Zhx2 participates in the regulation of bipolar cell subset fate determination during retinal development (64). Collectively, accumulating evidence demonstrated that ZHX2 is strongly involved in the developmental processes of different cells, which is consistent with the acknowledged ZHX2-mediated transcription of stemness genes. However, much work is required to better understand the exact roles and mechanisms of ZHX2 in organogenesis and tissue repair.

### ZHX2 and HBV infection

HBV is one of the well-known risk factors for HCC. According to the WHO (World Health Organization), almost one-third of the world’s population has been infected with HBV at some point in their lives (65, 66). HBV infects more than 250 million individuals worldwide, and almost 1 million die annually from complications of persistent infection, cirrhosis, and HCC (66).

As a liver cancer suppressor, ZHX2 expression is significantly decreased in tumor tissue from HBV-positive HCC patients and liver from HBV transgenic mice (48). Further studies show that HBV infection, especially the viral protein HBx reduces ZHX2 expression *via* upregulation of an oncomiR miR-155 (48) or CREB-mediated activation of miR-3188 (32), leading to liver cancer progression. In turn, ZHX2 serves as a novel restriction factor against HBV replication *via* regulating HBV promoter activities and cccDNA modifications. *In vitro* and *in vivo* experiments confirm that ZHX2 significantly inhibits HBc, HBsAg, and HBeAg expression (25), while overexpression of ZHX2 eliminates HBx-mediated proliferation of HCC cells (48). Mechanistically, ZHX2 binds to cccDNA and reduces the expression of histone regulator genes p300/CBP, leading to epigenetic repression of cccDNA (25). Alternatively, ZHX2 increases the expression of linc01431, a novel noncoding RNA for HBV restriction, which competitively binds with PRMT1 to block HBx-mediated degradation and enhances the occupancy of PRMT1 on cccDNA, thereby repressing cccDNA transcription (26). All in all, ZHX2 and HBV are mutually regulated by each other during HBV infection.

### ZHX2 and lipid metabolism

Interestingly, a study in mice using the QTL (quantitative trait locus) mapping strategy identified Zhx2 as a novel regulator of plasma levels of lipids, including triglyceride (TG) (23), indicating a potential role of Zhx2 in lipid metabolism. Compared with other mouse strains, BALB/cJ mice that harbor *Zhx2* defect exhibit decreased serum lipid levels and resistance to atherosclerosis when fed a high-fat diet (30). Constantly, altered hepatic transcript levels of several genes affecting lipid homeostasis, including *Lpl*, are detected in BALB/cJ mice (23). Notably, further research shows that ZHX2 inhibits the uptake of exogenous lipids in hepatocytes by transcriptional repression of LPL expression, which leads to cell growth retardation, and suppresses the progression of NAFLD to HCC (34). Concurrently, it has been found that ZHX2 increases transcription of miR-24-3p which binds to *SREBP1c* mRNA to promote its degradation, thereby inhibiting SREBP1c-mediated lipid *de novo* synthesis (24). The involvement of ZHX2 in fatty liver disease is further confirmed by a recent study showing that Zhx2 deficiency in hepatocytes exacerbates NASH progression by transcriptional activation of *Pten (* 37). Collectively, ZHX2 is a critical regulator of lipid metabolism, while we still need more studies to fully delineate the downstream network contributing to ZHX2-mediated lipid regulation.

### ZHX2 and immune regulation

ZHX2 is abundantly expressed in thymus and spleen (2), and increasing studies have shown that ZHX2 affects the development and function of different immune cells and participates in the progression of a variety of immune-related diseases.

#### ZHX2 is involved in the process of B-cell differentiation

A study using gene expression profiling describes an interesting expression pattern of *ZHX2* in B lymphoid cells. Similar to essential transcription factors *PAX5* and *E2A*, *ZHX2* is turned on during the transition from hematopoietic stem cells (HSCs) into early-B and shows a further increase in pro-B and later stages (27). Recently, Nagel et al. confirmed that ZHX2 is significantly upregulated in B cells while ZHX1 is downregulated. The reduced expression of ZHX2 together with the activation of ZHX1 may contribute to the deregulated B-cell differentiation phenotype in HL (67). However, to date, there were no reports about the role of ZHX2 in B cell development and functions. Interestingly, a genome-wide association study reveals rs10108684, the intronic SNP of *ZHX2*, as one of the eight top-scoring associations between SNPs and vaccinia antibody levels in African-Americans, strongly suggesting the critical involvement of ZHX2 in B cell-mediated antibody production (68). In summary, ZHX2 shows a dynamic expression pattern during B cell development but its function in B cell maturation is completely unknown and requires further studies.

#### ZHX2 inhibits NK cell maturation and function

Natural killer (NK) cells are primarily involved in innate immunity and possess important functional properties in anti-viral and anti-tumor responses (69–71). NK cells are derived from hematopoietic stem cells (HSC) *via* a series of developmental stages, including common lymphoid progenitor (CLP), NK cell precursors (NKP), immature NK cells and mature NK cells (72, 73) Multiple internal pathways and external factors contribute to the development of NK cells from HSCs (73). Tan et al. recently showed that ZHX2 significantly restricts the terminal maturation and effector functions of NK cells both *in vivo* and *in vitro (* 28). Mechanistically, ZHX2 controls NK cell maturation and function *via* two related pathways. ZHX2 down-regulates the responsiveness of NK cells to IL-15, the cytokine crucial for NK cell development and survival (74). On the other hand, ZHX2 controls the transcription of *Zeb2*, a transcription factor identified as a major driver of CD27^low^ NK cell maturation (75, 76). It has been reported that Zeb2 directly or indirectly modulates IL-15-mediated survival and development of NK cells (77, 78). Zeb2 might be associated with ZHX2-mediated regulation of IL-15 signaling (77, 78). Accumulation of immature NK cells has been reported in different tumors (79). The contribution of ZHX2 in the dysregulation of tumor-infiltrating NK cells strengthens ZHX2 as an immune checkpoint regulating NK cells. Targeting ZHX2 has great potential in NK cell-based cancer immunotherapy.

#### ZHX2 is a critical regulator of macrophages

Macrophages are a key subset of phagocytic cells that readily engulf and degrade dying/dead cells as well as invading bacteria and viruses (80). Macrophages are distributed widely in the body tissues and play a vital role in development, tissue homeostasis and repair, and immunity (81). Macrophages are highly plastic cells that usually present different polarization states in response to local milieu stimuli (82, 83). Recently, a computational network study indicates ZHX2 as one of the most regulated transcription factors in myeloid cells to avoid an avalanche transcription event (31) Our previous study showed that Zhx2 is an important transcription factor that regulates macrophage polarization *via* reprogramming macrophage glucose metabolism (29). *Zhx2* deletion in macrophages significantly attenuates systemic inflammation in mice, prolongs mice survival, attenuates pulmonary injury and reduces proinflammatory cytokines in septic mice (29). Specifically, loss of Zhx2 confers macrophage tolerance to LPS-induced sepsis, accompanied by reduced levels of pro-inflammatory cytokines and lactate release (29). Mechanistically, Zhx2 enhances the production of proinflammatory cytokines in macrophages by promoting glycolysis in a Pfkfb3-dependent manner (29). Accordingly, BALB/cJ strain mice are less likely to develop atherosclerosis, and this resistance to atherosclerosis can be repeated in BALB/c mice by the transfer of bone marrow-derived macrophages from BALB/cJ mice (30). That is, ZHX2 promotes macrophage survival and proinflammatory functions in atherosclerotic lesions (30). In addition, tumor-associated macrophages (TAMs) are critical modulators of the tumor microenvironment (84). The important role of ZHX2-mediated pro-inflammatory polarization of macrophages suggests that targeting ZHX2 to modulate TAM may be a promising strategy for anti-tumor immunotherapy.

## Conclusions and perspectives

As a transcription factor, ZHX2 transcriptionally regulates the expression of a series of genes that participate in cell proliferation, differentiation, and metabolism homeostasis. Accordingly, ZHX2 has a broader role in regulating multiple physiological and pathological processes, including cell development, immune regulation, cancer development, and metabolism-related diseases. Significantly, ZHX2 exerts its roles in a context-dependent manner. The exact mechanisms controlling the switch of ZHX2 function in health and diseases are still not clear. Nevertheless, it remains uncertain whether ZHX2 interacts with DNA directly or indirectly *via* other transcription factors to exert its transcriptional regulation role. Future research needs to be focused on ZHX2 structure, protein interactome, and high throughput screening to clarify its transcriptional regulation and identify new targeted genes. Equally important, the mechanisms that regulate ZHX2 expression are still uncertain. Accumulated studies have suggested that different stimuli regulate ZHX2 expression at different levels including transcription, post-transcription, and posttranslational modification levels. However, the mechanisms are not yet precisely understood. Moreover, in addition to hydroxylation, ubiquitination, and Nα-methylation, other PTMs that determine the biological function and nucleocytoplasmic shuttling of ZHX2 under different circumstances need to be further explored.

## Author contributions

NL, ZW, and CM designed and prepared the manuscript and the figures. CM gave guidance on the outline and revised the manuscript. All authors contributed to the article and approved the submitted version.

## Funding

This work was funded by grants from the National key research and development program (2021YFC2300603), the National Science Foundation of China (Key program 81830017, and 81902051), Taishan Scholarship (No.tspd20181201), Major Basic Research Project of Shandong Natural Science Foundation (No.ZR2020ZD12), Key Research and Development Program of Shandong (2019GSF108238).

## Conflict of interest

The authors declare that the research was conducted in the absence of any commercial or financial relationships that could be construed as a potential conflict of interest.

## Publisher’s note

All claims expressed in this article are solely those of the authors and do not necessarily represent those of their affiliated organizations, or those of the publisher, the editors and the reviewers. Any product that may be evaluated in this article, or claim that may be made by its manufacturer, is not guaranteed or endorsed by the publisher.
